# Atrial fibrillation detection in endurance athletes vs non-athletes: Positive predictive value and burden estimation using implantable cardiac monitors

**DOI:** 10.1016/j.hroo.2025.12.002

**Published:** 2025-12-10

**Authors:** Jon Magne Letnes, Andreas Berg Sellevold, Turid Apelland, Fedelix Phetogo Brown, Rune Byrkjeland, Marius Flasnes, Jan Pål Loennechen, Bjarne Martens Nes, Marius Myrstad, NEXAF Investigators

**Affiliations:** 1 Department of Circulation and Medical Imaging, Norwegian University of Science and Technology, Trondheim, Norway; 2 Clinic of Cardiology, St. Olavs University Hospital, Trondheim, Norway; 3 Department of Medical Research, Bærum Hospital, Vestre Viken Hospital Trust, Gjettum, Norway; 4 Department of Internal Medicine, Bærum Hospital, Vestre Viken Hospital Trust, Gjettum, Norway; 5 Department of Cardiology, University Hospital of North Norway, Tromsø, Norway

**Keywords:** Implantable cardiac monitors, Atrial fibrillation, Diagnostic accuracy, Sports cardiology, Cardiac devices, Arrhythmia detection

## Abstract

**Background:**

Implantable cardiac monitors (ICMs) enable continuous arrhythmia detection and quantification of atrial fibrillation (AF) burden. However, the influence of an athletic lifestyle on the positive predictive value (PPV) of ICM-detected AF episodes, and the accuracy of automated AF burden estimation, remain uncertain.

**Objective:**

This study aimed to compare the PPV of ICM-detected AF episodes between athletes and non-athletes with known AF, and to evaluate the accuracy of automatic AF burden estimation.

**Methods:**

During a 4-week pre-randomization phase of 2 randomized trials, 3041 AF episodes lasting ≥30 seconds were detected by ICMs in 32 endurance athletes and 159 non-athletes with non-permanent AF, totaling 4202 monitored days. All episodes were manually adjudicated to determine true AF.

**Results:**

The patient-averaged PPV was 0.59 (95% confidence interval [CI] 0.42–0.74) for athletes and 0.51 (95% CI 0.45–0.58) for non-athletes (*P* = .24). PPV increased significantly with longer episode duration, reaching 0.89 (95% CI 0.83–0.94) for episodes >60 minutes vs 0.42 (95% CI 0.35–0.48) for episodes <6 minutes. Findings were consistent across groups. Correlation between automatic and validated AF burden was 0.67 (95% CI 0.56–0.77), improving to 0.85 (95% CI 0.80–0.92) after excluding episodes shorter than 6 minutes.

**Conclusion:**

The PPV of ICM-detected AF episodes is similar in athletes and non-athletes. Longer AF episode duration enhances accuracy, and excluding short episodes improves automated AF burden estimation. ICMs may serve as good alternatives for AF monitoring also in athletic subpopulations.

## Graphical abstract

**Figure:**
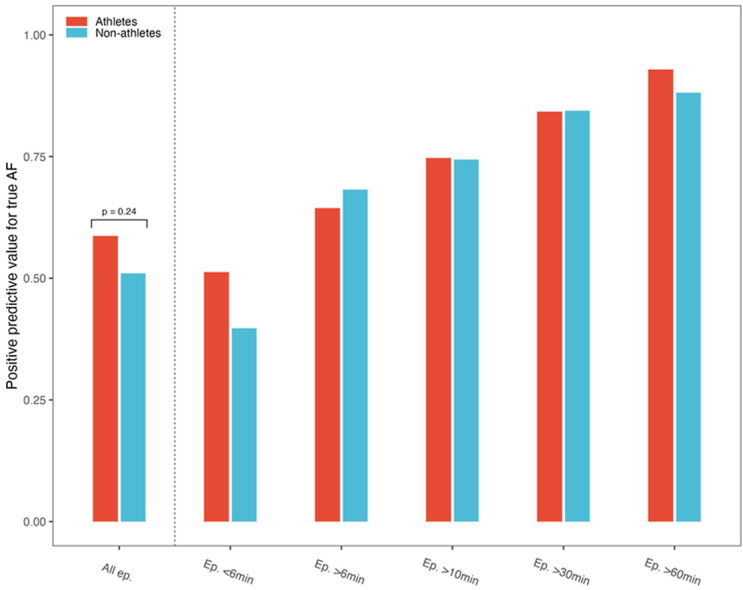

**Key Findings**

▪ The positive predictive value (PPV) of implantable cardiac monitor (ICM)-detected atrial fibrillation (AF) episodes was similar in endurance athletes and non-athletes.
▪ Longer AF episode duration was strongly associated with higher PPV—increasing from 0.42 for episodes <6 minutes to 0.89 for episodes >60 minutes.
▪ Automated (non-validated) AF burden estimates moderately correlated with validated AF burden (r = 0.67), improving substantially (r = 0.85) when episodes <6 minutes were excluded.
▪ These findings indicate that ICMs provide reasonable AF detection performance in both athletic and non-athletic populations, but excluding short-duration AF episodes enhances the accuracy of automated AF burden estimation and may improve clinical interpretation.

## Introduction

Over the last couple of decades, atrial fibrillation (AF) has evolved from solely being clinically detected on a surface electrocardiogram (ECG) to more commonly being detected through continuous monitoring from various medical and non-medical devices. These methods include clinician-initiated monitoring, such as pacemakers and implantable cardiac monitors (ICMs), but also patient-acquired and patient-initiated devices such as smart watches and ECG patches.^1^ With these changes in AF detection there has been a growing interest in the total time, or proportion of time, spent in AF (ie, AF burden) as a clinical predictor and marker for cardiovascular outcomes. A higher burden of device-detected AF has been linked to a higher risk of stroke and systemic embolism,^2^ and an increase in AF burden during follow-up has also been shown to predict imminent cardiovascular hospitalizations.^3^ Thus, AF has evolved from a dichotomous to a continuous measure. In line with this, a recent review highlighted the role of AF burden as an important treatment goal and a predictor of adverse cardiovascular outcomes with possible impact on cardiovascular risk stratification and treatment decisions.^4^ Validation of the technology used for quantifying AF burden was at the same time listed as a key knowledge gap for the further advancement in this field.

Although athletes generally are perceived to be healthier than the general population, they are prone to cardiac arrhythmia,^5^ and especially endurance athletes are known for increased risk of AF development.^6^ Arrhythmia monitoring is particularly challenging in athletes because of their very active lifestyle. Surface ECGs are prone to detached electrodes because ofbecause of perspiration and artifacts caused by extensive upper body movements during activity. Also, ICM recordings may be affected by certain physical activities. The sensitivity of ICMs for detecting AF episodes is consistently reported to be high at ≥90% across several studies,^7^, ^8^, ^9^, ^10^ but the positive predictive value (PPV) of automatically detected AF episodes has been reported to be highly variable^7^^,^^8^^,^^11^^,^^12^ and has not been previously studied in athletes. Furthermore, the accuracy of automatically detected AF burden is not established, which is of importance since adjudication of detected possible AF episodes is time-consuming in clinical practice. Detected AF episodes of shorter duration have been found to have lower PPVs for true AF,^13^ and taking this into consideration may improve accuracy of AF burden calculation.

Our aim for this study was to investigate PPV of ICM-detected AF episodes in highly physically active endurance athletes compared with non-athletes with AF and to describe differences in false-positive detections. We also aimed to assess the differential impact of AF episode length on PPV for these 2 groups, and to investigate the impact of AF episode length in the calculation of AF burden.

## Methods

### Study sample

We included data from patients with known paroxysmal or persistent AF from 2 ongoing randomized controlled trials (RCTs) investigating the interplay between exercise and AF in endurance athletes and non-athletes, respectively. The sample of athletes with AF was derived from the NEXAF Detraining study (ClinicalTrials.gov: NCT04991337), an international, multicenter RCT investigating the effects of training adaption on AF burden in endurance athletes with paroxysmal AF. For the current study, study participants at Bærum Hospital and St.Olavs Hospital, Norway, were included. Key inclusion criteria were age ≥18 years and reporting >5 weekly hours (running, rowing) or >8 weekly hours (cycling, cross-country skiing) of endurance sport practice. Key exclusion criteria were other cardiac, metabolic, and cardiovascular conditions.^14^ The sample of non-athletes were derived from the NEXAF Trial (ClinicalTrials.gov: NCT05164718), a multicenter RCT investigating the impact of a 1-year exercise intervention in previously non-athletic patients with paroxysmal or persistent AF. All study centers for the NEXAF Trial are found in Norway (St Olavs Hospital in Trondheim, Tromsø University Hospital of North Norway, and Bærum Hospital). The study finished inclusion in October 2024, with key inclusion criteria being age ≥18 and <80 years and reporting <150 minutes per week of moderate and/or <75 minutes of vigorous exercise the last 3 months. Exclusions were made based on the following criteria: permanent or long-standing AF, recent or planned AF ablation, unstable cardiovascular or pulmonary conditions, significant valvular or structural heart disease, severe comorbidities (eg, cancer, psychiatric, or cognitive disorders), contraindications to exercise, or circumstances influencing protocol adherence. Both depicted RCTs also required participants to have an ECG verified diagnosis of AF and recent symptomatic episodes of AF. Exhaustive details regarding inclusion and exclusion criteria are found in published protocol papers.^14^^,^^15^

All participants underwent standardized measurements of height, body mass, blood pressure, and resting heart rate following procedures denoted in the study protocols. Body mass index (BMI) was calculated. Information on type of clinical AF (paroxysmal and persistent) was decided based on review of medical records. The athletes logged all exercise sessions using personal sport watches, and all data was stored after transfer to a web-based platform (Fitrockr, Digital Rebels, Berlin, Germany or TrainingPeaks, Louisville, Colorado, USA). These data allowed for checking whether episodes of AF occurred during the time of exercise sessions. We did not have information regarding exercise sessions in the pre-randomization period for the non-athletes.

Ethical approval was obtained from the Regional Ethics Committee in Mid-Norway (ID 213848) and South-East Norway (ID 212748). All participants gave informed, written consent prior to study inclusion. The research reported in this paper adhered to the STROBE (STrengthening the Reporting of OBservational studies in Epidemiology) guidelines.

### ICM implantation and programming

A trained physician implanted an ICM (Confirm RX™, Abbott, Sylmar, California, USA) a minimum of 4 weeks prior to baseline clinical measurements and randomization. The device was implanted subcutaneously in the left parasternal position at the level of the third or fourth intercostal space depending on patient specific considerations. For men, implantation was done at a 45-degree angle and for women at a 10–15-degree angle from the sternum. After the procedure, R wave sensing was controlled, and in some instances the device position was adjusted for proper signal quality. The R wave amplitude at implantation was collected. Recommendations from the producer was followed for adjustment of dynamic range and maximal sensitivity. The ICM was connected wirelessly to the web-based merlin.net platform via the participants own smartphone using a dedicated mobile application (MyMerlin, Abbott). For the non-athletes the ICM was programmed to transfer all new episodes daily, but some participants with large numbers of episodes experienced episodes exceeding the storage capacity, meaning that some detected episodes did not have a recorded electrocardiogram (EGM). For the athletes, the ICM was programmed to transfer episodes every fourth week and thus the storage capacity was exceeded more often with a larger proportion of episodes missing an EGM for review.

The Confirm Rx AF detection algorithm tests for lack of regularity, presence of large variance of R-R intervals, and sudden onset of the detected episode. All 3 criteria must be met, and if these criteria are met, a P-wave discriminator is used by assessment of consistent P waves over 30 seconds. If consistent P waves are found the episode is rejected, and if not, the episode is stored as an AF episode.

### Validation procedure

All episodes classified as AF from the 4 weeks prior to randomization were included for adjudication, up to a limit of 50 episodes per participant. Episodes without a stored EGM were omitted. For 7 athletes, >50 episodes (58–167) were included, because of these episodes already being adjudicated in a previous study.

8 cardiologists (n = 5), hospital internists (n = 1), and cardiology residents (n = 2) performed the adjudication of episodes according to a predefined procedure. The merlin.net web portal gives access to an interactive “EGM gain viewer” allowing close-up evaluation of the EGM together with tools for precise measurement of cardiac intervals, and a scatterplot showing heart rate variability across time for the duration of the EGM. AF was classified as “true AF” or “false-positive AF”. In line with the definition of AF, episodes with an automatically detected duration >30 seconds on the EGM were rejected if the true EGM-verified duration was <30 seconds.-False-positive AF episodes were further classified with ≥1 of the following findings (1: artefact, 2: atrial flutter, 3: supraventricular tachycardia, 4: premature supraventricular beats, 5: premature ventricular beats, 6: sinus arrhythmia, 7: pause, 8: other). Reviewers were also instructed to comment on issues of over- and under-sensing. Each episode was adjudicated by 1 reviewer, but if the uncertainty after evaluation of the AF episode was more than minimal regarding the presence of true AF, the episode was marked as “uncertain” and reviewed a second time by one of the other reviewers blinded for the first reviewer’s evaluation. In case of a tie after the second review, a third, blinded review was performed by a cardiac electrophysiologist with extensive experience in managing cardiac arrhythmias, resolving the tie.

### Statistics

The patient-averaged PPV was calculated as the number of true positive AF episodes divided by-the sum of true positive and false-positive AF episodes for each individual and then averaged across individuals. This was done to reduce inflation of results from individuals with large numbers of episodes. PPVs were calculated for athletes and non-athletes separately and for both groups combined for all episodes and by duration of episodes (<6 minutes, >6 minutes, >10 minutes, >30 minutes, >60 minutes, and >24 hours). Cut-offs for AF episode length were chosen based on cut-offs from previous studies allowing for comparison of results.^13^^,^^16^ The total PPV was calculated as the number of true positive AF episodes divided by the sum of true-positive and false-positive AF episodes. We also calculated patient-averaged PPVs by sex, BMI (over/under 30 kg/m^2^), age groups (age <50, 50 to 70, >70 years), and ICM R wave amplitude (<0.25, 0.25–0.54, 0.55–0.69, 0.70–1.2, and >1.2mV). 95% confidence intervals (95% CIs) were constructed by bootstrapping using the percentile method with 500 iterations. AF burden was calculated based on all included episodes. For those having >50 episodes within the 4-week study period, we used the duration from the first to the last adjudicated episode as the time under observation, whereas for patients with ≤ 50 episodes we used the whole 4-week period as the time under observation. To address the impact of AF episode length on AF burden estimates we also calculated AF burden including only episodes lasting >6 minutes and 60 minutes, respectively. Correlation analyses between measures of AF burden were performed by Spearman’s correlation because of a non-normal data distribution. The Cohen’s Kappa for agreement between 2 independent reviewers was calculated for AF episodes classified as “uncertain” (ie, AF episodes most difficult to classify) in first round of review after repeated review by a second reviewer. Statistical comparisons across groups for descriptive variables were performed by the χ2 test; Fisher’s exact test; Mann-Whitney U test or Student’s *t* test as appropriate based on the data distribution. All statistical analyses were performed using R version 4.4.2 (2024-10-31; www.r-project.org).

## Results

**Figure 1:**
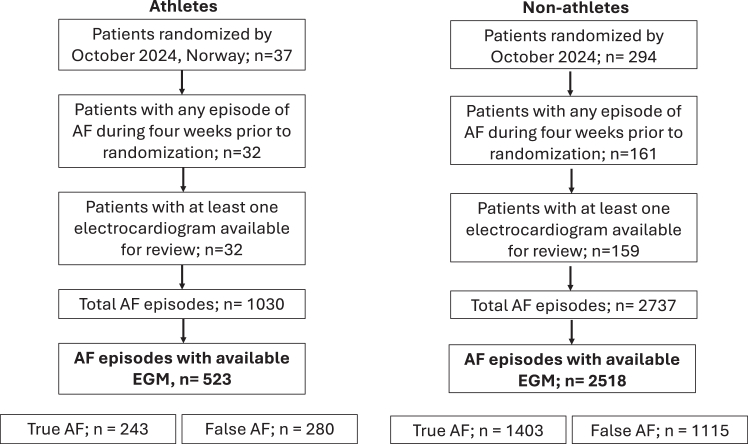
Overview of study participants and included AF episodes.

Overall, 32 athletes and 159 non-athletes had at least 1 detected AF episode during the 4 weeks prior to randomization and an EGM available for review. The total time monitored was 4202 days (mean 22, standard deviation [SD] 10), and 3041 episodes were included for review. A flowchart with overview of included participants and episodes are shown in Figure 1. Key baseline characteristics are shown in Table 1.

**Table 1:** General characteristics

	Athletes	Non-athletes	*P*-value
	N = 32	N = 159	
Age, years	57 (±9)	65 (±10)	<.001
Sex, male	29 (91%)	107 (67%)	.008
AF type			.002
Paroxysmal	32 (100%)	118 (75%)	
Persistent	0 (0%)	39 (25%)	
Any heart failure	0 (0%)	7 (4.4%)	.60
Myocardial infarction	0 (0%)	12 (7,5%)	.20
Stroke or TIA	0 (0%)	7 (4.4%)	.60
BMI, kg/m^2^	23.9 (±2.5)	27.4 (±4.7)	<.001
Systolic BP, mm Hg	128 (±13)	134 (±16)	.036
Diastolic BP, mm HG	76 (±9)	81 (±16)	.014
Resting heart rate, bpm	49 (±7)	61 (±10)	<.001
R wave amplitude, mV	0.58 (±0.20)	0.53 (±0.24)	.30
Crude AF burden, %	1.6 (1.8)	5.2 (10.2)	.75
Validated AF burden, %	1.0 (1.6)	3.9 (9.2)	.105

Mean (±SD); n (%).

AF = atrial fibrillation; BMI = body mass index; BP = blood pressure; TIA = transient ischemic attack.

**Figure 2:**
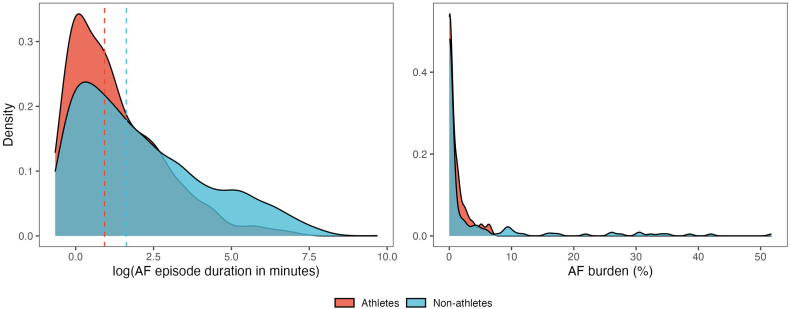
Distribution of AF episode duration and AF burden for athletes and non-athletes. The dotted lines represent median AF episode duration.

After adjudication 25 athletes (78%) and 100 non-athletes (63%) had at least 1 episode of true AF; 20 athletes (63%) and 92 non-athletes (58%) of these, respectively, had at least 1 AF episode with duration >6 minutes. Furthermore, 13 athletes (41%) and 77 non-athletes (48%) had an AF episode with duration >60 minutes. The median AF episode duration was shorter in athletes (median 2.5, interquartile range [IQR] 1.1 to 9.4 minutes) compared with non-athletes (median 5.2, IQR 1.6 to 31.1 minutes) (Figure 2), and heart rate during AF episodes was significantly lower (mean 76 [SD 19] vs 96 [SD 30] bpm, *P <* .001).

### Positive predictive value of AF episodes

**Figure 3:**
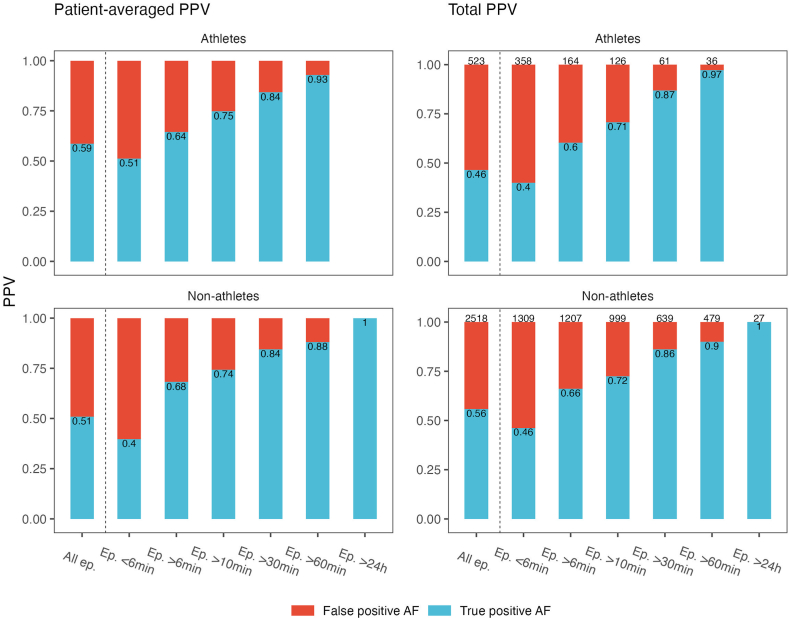
Patient-averaged PPV and total PPV for athletes and non-athletes across duration of AF episodes. The number of episodes in each category is given on top of each column for the total PPV panel, and the exact PPVs are given on each column for both panels. AF = atrial fibrillation; BMI = body mass index; PPV = positive predictive value.

Across all episodes the patient-averaged PPV was 0.53 (95% CI 0.46–0.59) and the total PPV was 0.54 (95% CI 0.52–0.56). There were no significant differences between athletes and non-athletes for the patient-averaged PPV. However, the total PPV was lower in athletes (0.46, 95% CI 0.42–0.50) compared with non-athletes (0.56, 95% CI 0.54–0.58). This was mainly because of a larger proportion of AF episodes with a short duration among athletes and a lower PPV for episodes <6 minutes duration. For all other AF episode durations, the PPV was similar for athletes and non-athletes (Figure 3). Owing to the low number of women in the group of athletes, we also performed analyses excluding women, but the results did not change significantly between athletes and non-athletes. The same impact on episode duration was also found across sex.

**Figure 4:**
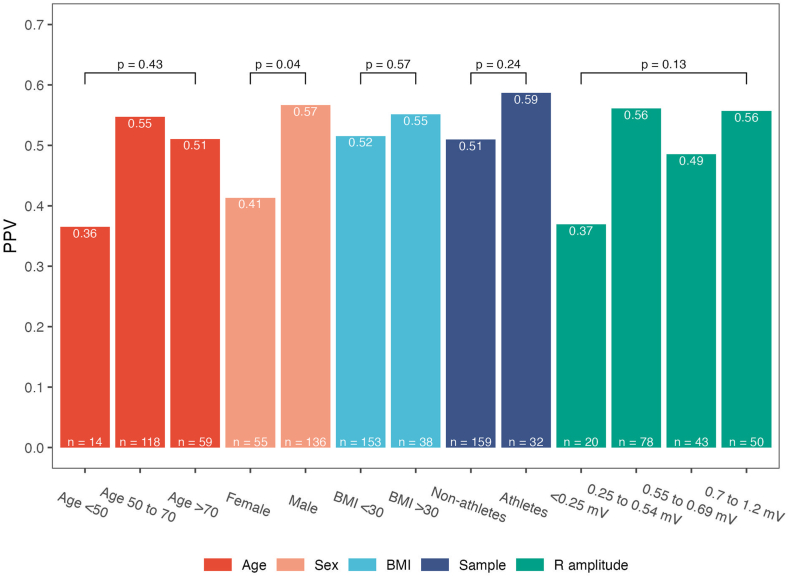
Patient-averaged positive predictive value (PPV) by various clinical factors. BMI = body mass index; PPV = positive predictive value.

Episodes of longer duration had a higher PPV compared with the shorter episodes in a dose-response manner. The patient-averaged PPV with 95% CIs by AF episode duration is shown in Supplemental Table 1 and PPVs are displayed graphically in Figure 3. The number of episodes lasting >24 hours was low (n = 27), but all episodes represented true positive AF (PPV of 1.00). All these episodes occurred in non-athletes. The impact of age, sex, BMI, athletic status, and R wave amplitude on patient-averaged PPVs is shown in Figure 4. No significant correlation was found between PPV and R wave amplitude (Spearman rho 0.07, *P* = .36). The only significant difference in PPV across these groups were found for men compared with women (PPV 0.57 vs 0.41, *P* = .04), with men having higher R wave amplitudes than women (0.46 mV vs 0.57 mV), and 16% of women had R wave amplitudes <0.25 mV compared with 8% of men.

Reasons for misclassification of AF is shown in Table 2. Premature atrial contractions and artifact/other dominated as the cause of misclassification and the latter was more prevalent in athletes. Artifacts and other causes included issues like T wave over-sensing and QRS under-sensing. The athletes had less misclassifications because of premature ventricular contractions but a higher proportion of sinus arrhythmia.

**Table 2:** Findings for false positive AF episodes

Finding	Athletes (n = 280)∗	Non-athletes (n = 1115)∗	*P*-value
Atrial flutter	8 (2.9%)	46 (4.1%)	.3
Other supraventricular tachycardia	0	4 (0.4%)	.6
Premature atrial contractions	115 (41%)	496 (44%)	.3
Premature ventricular contractions	8 (2.9%)	100 (9%)	<.001
Sinus arrhythmia	33 (12%)	43 (3.9%)	<.001
Pause	0	3 (0.4%)	>.9
Artefact/other	123 (44%)	309 (28%)	<.001

Numbers are n (%).

∗ Number of false-positive AF episodes.

The lower PPV in younger individuals (Figure 4) was associated with a relative overweight of sinus arrhythmia causing false positives in the youngest age group. Sinus arrhythmia was found in 21% of the false positives from this age group compared with 7% of the 2 other age groups.

81 AF episodes was detected during exercise for the athletes. 38 of these lacked an EGM, with 9 true positive AF episodes for the remaining 43 (PPV 0.21). The mean duration of these episodes was 12.8 minutes (SD 17.3 and range 0.7 to 71.4 minutes). Dominating causes of misclassification of the 34 false-positive AF episodes was artifacts (n = 24, 71%) and other not classified reasons (n = 7, 21%) caused by noise during activity and R wave under-sensing because of low R amplitudes. The latter possibly because of increased ventilatory movements during vigorous activity.

A total of 125 (4.1%) episodes was classified as “uncertain” after the first round of review and re-reviewed by a second reviewer. After second round of review agreement was made in 84 of 125 episodes (Cohen’s Kappa 0.38, *P <* .001, signifying fair agreement), with 41 ties resolved by a third reviewer.

### Quantification of AF burden

The distribution of validated AF burden for athletes and non-athletes is shown in Figure 2. Across athletic status the crude (ie, not validated) AF burden was mean 4.6% and median 0.8%, whereas the AF burden based on validated episodes was mean 3.4% and median 0.002%. Excluding episodes <6 minutes duration, the crude and validated AF burden was mean 3.3% and 2.7%, and median 0% for both. The Spearman correlation coefficient between crude and validated AF for all episodes was 0.67 (95% CI 0.56–0.77, *P <* .001). Including only episodes >6 minutes, the correlation was 0.86 (95% CI 0.80–0.92, *P <* .001), and including only episodes >60 minutes the correlation was 0.94 (95% CI 0.89–0.99, *P <* .001). These results were similar in separate analyses for athletes and non-athletes. Although AF episodes <6 minutes duration represented 45% of all episodes, they only represented 1.4% of the crude AF burden, and 0.6% of the validated AF burden.

## Discussion

This study shows that the PPV for ICM-detected AF episodes was acceptable but moderate, and an increasing episode duration was associated with significantly higher PPVs. Overall, PPVs were similar in athletes and non-athletes. Both in athletes and non-athletes, PPVs were low for episodes with a duration <6 minutes. Omitting episodes lasting <6 minutes improved the correlation between validated and non-validated measures of AF burden. The similar PPVs for athletes and non-athletes suggest that ICMs may serve as good alternatives for AF monitoring, also in athletic subpopulations. It should be noted that the PPV for episodes occurring during exercise was markedly lower, although the limited number of episodes limits the certainty of these findings.

Our results reinforce the findings from other studies of PPVs for ICM-detected AF using ICMs of various types. A large study was recently published by Gala et al^13^ on data collected in clinical practice from 16,230 AF episodes, lasting at least 6 minutes, detected by the Confirm Rx (Abbott) ICM. They reported a patient-averaged PPV of 0.67 for episodes >6 minutes, which is practically identical to our findings. One of our key findings is the profound impact of episode duration on PPVs both for athletes and non-athletes. Similarly, the study by Gala et al^13^ reported a significant impact of AF episode duration with patient-averaged PPVs of episodes >60 minutes duration being 0.83, which is slightly lower compared with our findings (patient-averaged PPV of 0.89). In a study using the Medtronic Reveal LINQ ICM, a sample of 1049 patients with known AF had 16,506 episodes of AF over 2 minutes duration detected, and the patient-average PPV was 0.73. For episodes >6 and 60 minutes the PPV was 0.81 and 0.95, respectively. Other studies, including a lower number of AF episodes (86–1689 episodes) has reported PPVs ranging from 0.38 to 0.82.^7^^,^^8^^,^^11^^,^^12^ There may be small differences in PPVs across ICM vendors, but before direct comparisons are made in populations with similar inclusion criteria, one should be careful about making conclusions. Also, it could be argued that a reduced PPV because of a higher sensitivity in trade-off with a lower specificity may be a relevant trade-off as false-negatives may be more problematic for patient management.

The higher total PPV for all episodes for non-athletes compared with athletes were explained by differences in the distribution of AF episodes, which was evident in analyses by patient-level PPVs across AF episode duration. The difference in sex distribution between the 2 populations did not explain these differences as men had higher PPVs, and the athletic group had a higher proportion of men.

Reflecting the moderate PPVs across most studies there is obvious gains to be made for algorithms to detect and classify AF. For example, the use of artificial intelligence may have the potential to relieve physicians of time-consuming work.^17^ In line with this, 1 study investigated the impact of employing a deep neural network for reducing the amount of false-positive AF episodes.^11^ Using the novel approach the PPV increased from 0.54 to 0.75. As could be expected from our findings, the gain was the highest for shorter duration episodes. It should be noted that the PPVs should be expected to vary based on the populations studied and true prevalence of AF.

The only significant impact on PPV was found for sex, with women having significantly lower PPVs than men, and a previous study using the same type of ICM reported similar findings.^13^ The finding of lower R wave amplitudes for women suggests that differences in anatomy and body composition, together with a theoretically smaller cardiac mass, may play a role. Thus, we also expected obesity (BMI ≥30 kg/m^2^) to have an impact on PPVs, but there was no significant difference with the PPV being numerically higher in the obese group. There was a strong numerical trend toward lower PPV in patients with R wave amplitudes <0.25 mV; however, the results did not suggest a further dose-response effect toward increasing PPVs with higher R wave amplitudes.

### Importance for research and clinical work

These results suggest that the Abbott Confirm ICM provide acceptable PPVs for AF detection both for endurance athletes and non-athletes. However, based on the impact of R wave amplitudes in our study, we recommend considering repositioning ICMs if R wave amplitudes are lower than 0.25 mV, even though the reported differences did not reach statistical significance.

As demonstrated in our study, athletes seem to have lower AF burden and often present with short AF episodes, suggesting prolonged monitoring may be necessary to detect paroxysmal AF and ICM as an attractive alternative in this group. Although there was an issue with low PPV of detected episodes during exercise, these issues would be expected to also impact other external methods for ambulatory monitoring such as patch ECGs^1^ and surface ECGs. Practical issues, such as surface electrodes losing contact because of sweating and movements, are not relevant for ICMs, and ICMs are usually also well-tolerated. However, costs related to implantation and equipment may limit its use, and there seems to be a need to refine algorithms for AF detection during exercise, at least based on this limited data. This aligns with previous considerations that the accuracy of a device is expected to vary depending on algorithm, patient population, and monitoring setting.^18^

The impact of shorter episodes on the total burden of AF is low. Still, as some patients have low burden, short episodes may impact the burden estimates at individual level. Thus, when interested in patient-level data and change in AF burden within patients, one could consider omitting or validating episodes with a duration <6 minutes. In support of a reduced attention toward AF episodes of short duration, is observational findings from the CIRCA-DOSE study.^19^ There, the clinical importance of short-duration episodes of AF was questioned given that longer episode duration (and higher AF burden) was associated with more health care utilization and lower quality of life. The impact of episode duration on AF related quality of life in athletes with AF has recently been reported.^20^ The impact of AF episode duration on clinical end points such as stroke is not yet established in athletes, but a diagnosis of AF has been shown to increase stroke risk both in athletes and non-athletes.^21^^,^^22^ All in all, there may be a rationale for omitting review of short duration AF episodes (eg, <6 minutes) detected from ICMs to improve resource utilization and probably without negative impact on patient management.^23^

Estimates of AF burden from a series of long-duration episodes is more trustworthy than multiple short-duration episodes, with the latter requiring manual review for diagnostic accuracy to be adequate.

Consumer-initiated use of wearables (eg, ECG- or photoplethysmography-based smartwatches) allowing detection of AF and clinician-initiated devices for arrhythmia screening (eg, ECG-patches) are increasing in use, and each method has strengths and limitations as previously outlined.^18^ As we are still in the very beginning of understanding the clinical impact and meaning of arrhythmias detected from continuous monitoring, the growing use of wearables challenges us in interpreting findings from such recordings. Conversely, because of the invasive nature and lack of data supporting screening for AF with ICMs, the use of ICMs for AF/arrhythmia monitoring should probably be limited to clinical settings were quantification or detection of AF will have clear consequences for patient management.

### Strengths and limitations

Strengths of the current study includes prospectively gathered data following standardized procedures from 2 RCTs on patients with AF. The current study used only 1 type of ICM, and caution should be made for generalizing the findings to other ICM types and methods for AF burden quantification. However, studies using the Medtronic Reveal have also found that episode duration impacts PPVs, and it is very likely that these findings are applicable across different ICM vendors. For the athletes, the number of detected AF episodes occurring during logged exercise sessions were low. Although this study focused on the PPVs from AF episodes, we regret we did not have data on the sensitivity of AF detection during the monitoring period. Another limitation was the considerable number of missing EGMs for the athletes because of longer transmission interval. However, there were still a considerable number of included episodes, and the findings were very similar across athletes and non-athletes. Other studies are warranted to assess the PPV of ICMs to detect other cardiac arrhythmias.

## Conclusion

The PPV of ICM-detected AF episodes is similar for athletes and non-athletes, and the PPV is significantly influenced by episode duration with low PPVs for short-duration episodes and high PPVs for longer duration episodes. Excluding short episodes enhances the accuracy of automated AF burden estimation. These findings emphasize the importance of adjudicating shorter episodes, or considering excluding them, to improve the reliability of AF burden assessments.

## Disclosures

The authors have no conflicts of interest to disclose.

## Footnotes

Appendix Supplementary data associated with this article can be found in the online version at https://doi.org/10.1016/j.hroo.2025.12.002.
