# Supplementary material for: Atrial fibrillation detection in endurance athletes vs non-athletes: Positive predictive value and burden estimation using implantable cardiac monitors
Source: Heart Rhythm O2. 2025 Dec 10;7(2):284–92. doi: 10.1016/j.hroo.2025.12.002 (PMC12925923; doi:10.1016/j.hroo.2025.12.002)
Supplement: Supplementary Table [file mmc1.doc]

Supplementary Table 1. Mean patient-averaged positive predictive value with 95% confidence intervals (CI).

| Episodes | Athletes | Non-athletes | Combined |
| --- | --- | --- | --- |
| All | 0.59 (95% CI 0.42-0.74) | 0.51 (95% CI 0.45-0.58) | 0.53 (95% CI 0.46-0.59) |
| <6min | 0.51 (95% CI 0.35-0.67) | 0.40 (95% CI 0.33-0.47) | 0.42 (95% CI 0.35-0.48) |
| >6min | 0.64 (95% CI 0.47-0.81) | 0.68 (95% CI 0.61-0.76) | 0.68 (95% CI 0.61-0.74) |
| >10min | 0.75 (95% CI 0.58-0.90) | 0.74 (95% CI 0.66-0.82) | 0.75 (95% CI 0.68-0.81) |
| >30min | 0.84 (95% CI 0.68-0.96) | 0.84 (95% CI 0.78-0.91) | 0.84 (95% CI 0.78-0.90) |
| >60min | 0.93 (95% CI 0.79-1.00) | 0.88 (95% CI 0.82-0.94) | 0.89 (95% CI 0.83-0.94) |
| >24h | - | 1 | 1 |
